# Transformer Incipient Fault Prediction Using Combined Artificial Neural Network and Various Particle Swarm Optimisation Techniques

**DOI:** 10.1371/journal.pone.0129363

**Published:** 2015-06-23

**Authors:** Hazlee Azil Illias, Xin Rui Chai, Ab Halim Abu Bakar, Hazlie Mokhlis

**Affiliations:** 1 Department of Electrical Engineering, Faculty of Engineering, University of Malaya, 50603 Kuala Lumpur, Malaysia; 2 UM Power Energy Dedicated Advanced Centre (UMPEDAC), Level 4, Wisma R&D UM, University of Malaya, Jalan Pantai Baharu, 59990 Kuala Lumpur, Malaysia; Beihang University, CHINA

## Abstract

It is important to predict the incipient fault in transformer oil accurately so that the maintenance of transformer oil can be performed correctly, reducing the cost of maintenance and minimise the error. Dissolved gas analysis (DGA) has been widely used to predict the incipient fault in power transformers. However, sometimes the existing DGA methods yield inaccurate prediction of the incipient fault in transformer oil because each method is only suitable for certain conditions. Many previous works have reported on the use of intelligence methods to predict the transformer faults. However, it is believed that the accuracy of the previously proposed methods can still be improved. Since artificial neural network (ANN) and particle swarm optimisation (PSO) techniques have never been used in the previously reported work, this work proposes a combination of ANN and various PSO techniques to predict the transformer incipient fault. The advantages of PSO are simplicity and easy implementation. The effectiveness of various PSO techniques in combination with ANN is validated by comparison with the results from the actual fault diagnosis, an existing diagnosis method and ANN alone. Comparison of the results from the proposed methods with the previously reported work was also performed to show the improvement of the proposed methods. It was found that the proposed ANN-Evolutionary PSO method yields the highest percentage of correct identification for transformer fault type than the existing diagnosis method and previously reported works.

## Introduction

Power transformer is one of the important equipment in power systems since the transformer is vital to step-up or step-down the voltage and isolation of the electrical power. Thus, transformer breakdown may interrupt the power systems. The transformer fault leads to the electrical and thermal stresses, which will eventually cause the breakdown of insulating materials and the release of gaseous decomposition products respectively. Corona, sparking, arcing and overheating are subject to fault related gases released including hydrogen (H_2_), methane (CH_4_), acetylene (C_2_H_2_), ethylene (C_2_H_4_), ethane (C_2_H_6_) and carbon monoxide (CO) [1, 2].

Thus, transformer maintenance is very important and proper monitoring on the transformer condition will help to avoid the breakdown of transformer. Transformer oil condition monitoring is one of the fundamental methods in maintaining power transformers. The oil test types can be categorised into physical, chemical, electrical and environment type. Among all the tests, the chemical test, dissolved gas analysis (DGA) is commonly used which diagnose the faults based on certain ratio of dissolved gas in oil sample [3]. The existing methods of DGA are key gas method, Doenernberg’s ratio method, Roger’s ratio method and IEC method.

DGA methods involve processing data of the transformer oil sample and fault recognition through annalist experience and ability. However, the main problems with the existing DGA methods are it relies heavily on the experts and the actual site testing has shown that different DGA methods lead to different fault type. Hence, research on reliable techniques to diagnose the transformer fault is actively ongoing.

Since the past, there have been many works conducted on the applications of artificial intelligence and optimisation in condition monitoring on power system components and fault diagnosis, including transformer incipient fault diagnosis [4–10]. These include artificial neural network (ANN), fuzzy logic, rough set theory, support vector machine (SVM) and genetic programming.

One of the most widely used artificial intelligence methods in transformer fault prediction is artificial neural network (ANN) [1, 11, 12]. ANN is widely used due to it can learn from the training data directly and the complexity of computation in ANN is less. It is also adaptive, able to handle various nonlinear relationships and can generalize solutions for a new data set [13]. ANN directly implements the association process of inputs, where for transformer incipient fault prediction, it is the gas concentration and the outputs or fault type. Hence, physical model and a predefined correspondence function are not required. However, the convergence is slow and sometimes oscillation occurs. Also, the parameters of the ANN, such as the number of neuron and hidden layer, must be properly chosen in order to obtain the best performance of the network.

Many researches have been performed on the use of ANN in DGA methods to facilitate the detection of transformer incipient fault [11, 14]. The input and output from the DGA results of transformer oil were used to train a neural network and identify the fault type from the trained network. Although the use of ANN in transformer incipient fault detection seems to be reasonable, the chosen ANN parameters might not yield the best accuracy of the network output.

In one of the previous works, ANN was combined with the knowledge based of expert system for transformer fault diagnosis from DGA analysis [4]. The combination of both methods yields better performance than each method being used individually. This is due to the combination of the ANN and expert system takes advantage of superior features of each method and allows them to dominate different fault diagnosis. The usage of fuzzy logic has shown that the fault type of transformer can be obtained efficiently [15]. Fuzzy logic was applied as practical representation of the relationship between the gas content levels and fault type and with fuzzy membership functions. A combination of three fuzzy methods shows that the accuracy of the method is higher than a single fuzzy system in identifying the transformer fault type.

Combination of Artificial Immune System (AIS) and ANN was proposed in [7] to assess the transformer fault type based on DGA analysis. The AIS was used to determine the centers of the Radial Basis Function Neural Network (RBFNN). It was shown that the combination of AIS and RBFNN yields better transformer diagnosis accuracy than random selection and k-means clustering in determining the RBFNN hidden centers. Other neural network application has also been employed to improve the diagnostic accuracy of power transformer fault classification based on DGA analysis. Bootstrap and genetic programming (GP) feature extraction were combined with ANN and KNN classifiers [5]. The bootstrap eliminated the less fault type samples in the DGA data. Then, the features of the DGA data extracted with GP were used as the inputs in ANN and KNN classifiers. It was reported that bootstrap and GP combined with KNN yields a higher accuracy of transformer fault classification.

Genetic wavelets network (GWN) was proposed to enhance the existing DGA methods for transformer incipient fault identification [8]. The method combined genetic algorithm (GA), wavelet and ANN. GA was used to determine the optimal parameters of GWNs to achieve the best diagnostic DGA model. The wavelet transform property and the decomposed data feature extracted important information from the input data. It was reported that the proposed GWN method yields the best classification for the transformer fault identification compared to without wavelet transform.

Self-organizing polynomial networks (SOPN) was proposed as an intelligent decision making for the transformer fault diagnosis [9]. In this technique, the problem is heuristically formulated into a hierarchical architecture with several layers of simple low-order polynomial functional nodes. The networks handled the complicated and uncertain relationships of DGA data from transformer oil samples. The work reported that the proposed method yields far superior performance than the conventional DGA and ANN classification methods.

Although many previous works have reported on the use of intelligence methods to predict the transformer oil faults, it is believed that the accuracy of the previously proposed methods can still be improved. Since artificial neural network (ANN) and particle swarm optimisation (PSO) techniques have never been reported in the previous literature, a combination of ANN and various PSO techniques to predict the transformer incipient fault are proposed in this work. The advantages of PSO are simplicity and easy implementation. In this work, the possibility of using various particle swarm optimisation (PSO) techniques with ANN in identifying the transformer incipient fault is explored.

PSO is an evolutionary algorithm that is widely implemented in optimisation problems [16–19]. The thought process behind the algorithm was inspired by the social behaviour of animals, such as bird flocking or fish schooling [20–23]. PSO is a population based search algorithm characterized as conceptually simple, easy to implement, computationally efficient, rapid convergence and has the ability to avoid the local minima in a successful way. Hence, these characteristics are advantageous to complex optimisation problems which use huge number of parameters and have difficulty in obtaining the analytical solutions.

In this work, the PSO optimisation methods used are conventional PSO method, iteration PSO (IPSO) and evolutionary PSO (EPSO) method. The percentage of correct prediction of transformer incipient fault from the proposed methods was compared with each other and also with the methods which use only ANN technique and the existing DGA technique. The results from the proposed methods were also compared with the previously reported work to show the improvement of the proposed methods. Hence, the best type of PSO method combined with ANN could be identified, which may improve the transformer incipient fault diagnosis.

This paper is presented as follows. In section 2, the implementation of ANN is described. Section 3 explains various methods of PSO used in this work. They include PSO, iteration PSO (IPSO) and evolutionary PSO (EPSO). Section 4 discusses all results obtained from each method. The section includes results from the application of various PSO techniques and ANN, ANN alone and an existing DGA method in predicting the transformer incipient fault. Finally, section 5 summarises all findings obtained from this work.

### Artificial Neural Network (ANN)

ANN is a computational model to imitate the biological neural networks, which consists of interconnected neurons in order to compute the output from the input. ANN is widely used due to its ability to interpolate and extrapolate from the experience of analysing the data and able to reveal highly nonlinear input-output relationship [24]. Since the condition of electrical systems changes, ANN can adapt itself to new state and put the new state into new training [25]. Hence, ANN is a good approach to compute the relationship, which is difficult to describe explicitly. In this work, the neural network was developed in MATLAB programming language.

### Input and output data

**Fig 1:**
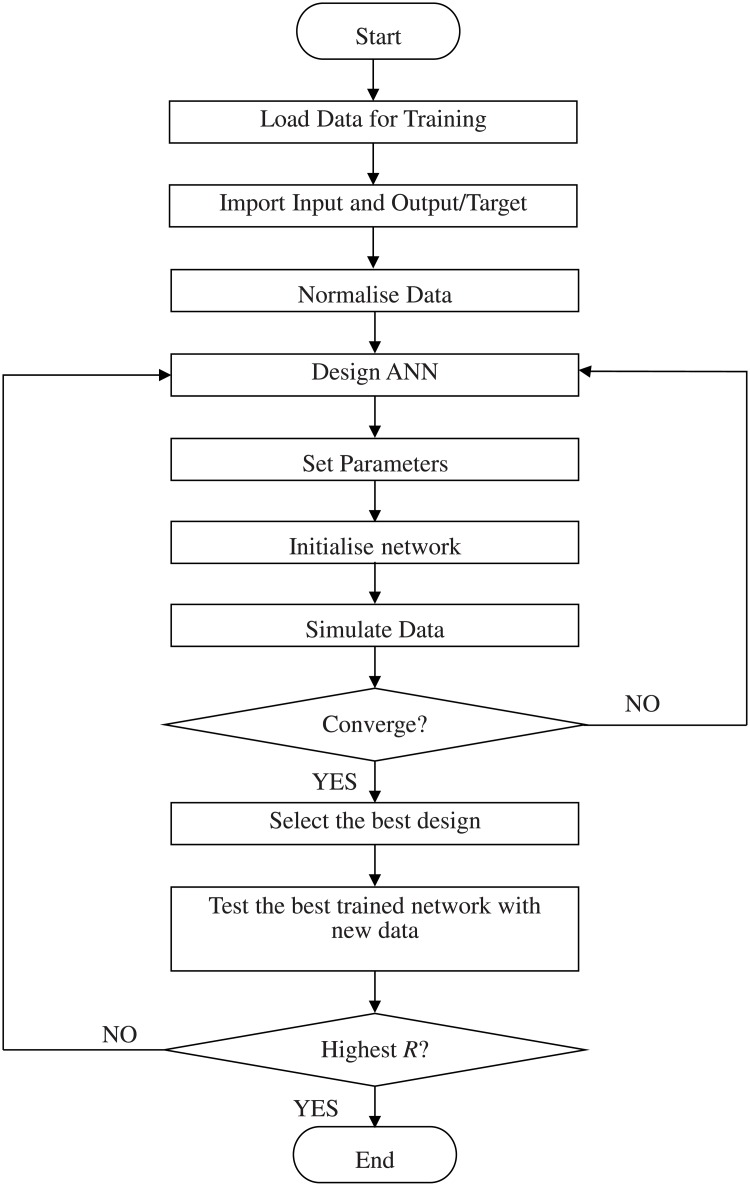
Flowchart of ANN algorithm.

In designing ANN, the selection of input, output and network topology is subject to performance of the ANN model [26]. The data of the gas compositions with respect to the incipient transformer fault were obtained from the actual data of an electrical utility. The design of ANN can be divided into two stages; the training and testing. The flowchart of the development of ANN is shown in Fig 1. Table 1 shows some of the actual data of incipient transformer fault from an electrical utility that were used in this work.

**Table 1:** Some actual data of incipient transformer fault from an electrical utility.

H_2_	CH_4_	C_2_H_2_	C_2_H_4_	C_2_H_6_	CO	Fault Type
4566	671	683643	434322	45482	2001	High intensity discharge
2323	782	545454	342233	4343	4545	High intensity discharge
2118	844	540711	449264	4443	4535	High intensity discharge
2285	706	546779	435718	4303	4235	High intensity discharge
2238	826	537988	335279	4008	4472	High intensity discharge
2373	817	669150	447061	4284	4807	High intensity discharge
2394	754	673175	360327	4049	4964	High intensity discharge
2423	765	535231	305712	4266	4523	High intensity discharge
2127	825	595394	369165	4456	4931	High intensity discharge
2400	774	647129	315114	4462	4274	High intensity discharge
9750	720	40	11	220	951	Low intensity discharge
9619	780	38	9	220	904	Low intensity discharge
9693	702	38	10	198	928	Low intensity discharge
9439	700	35	10	233	902	Low intensity discharge
9201	744	40	12	226	935	Low intensity discharge
9704	719	39	11	208	982	Low intensity discharge
9823	707	39	8	231	965	Low intensity discharge
9531	744	36	8	220	993	Low intensity discharge
9840	788	37	9	225	991	Low intensity discharge
9032	785	36	12	219	939	Low intensity discharge
3872	6008	2	21315	4772	6811	Thermal fault
4390	5843	3	21102	4474	6894	Thermal fault
3883	5578	2	24716	4797	6947	Thermal fault
4304	6135	5	24999	4508	6648	Thermal fault
4410	5750	5	24560	4630	6988	Thermal fault
3908	5587	5	20537	4315	6658	Thermal fault
3660	5862	2	20487	4590	6715	Thermal fault
3923	6273	5	22803	4624	6759	Thermal fault
4077	6036	2	23232	4469	6710	Thermal fault
4133	5902	4	20449	4608	6772	Thermal fault
4051	5507	3	22825	4330	6599	Thermal fault
4262	5856	2	22286	4689	6825	Thermal fault
200	1000	800	200	875	40	No fault
0	100	3.22	90	0	100	No fault
0	0	0	0	0	0	No fault
0	0	100	0	150	40000	No fault
600	400	280	400	250	300	No fault
600	450	300	800	400	300	No fault
300	50	14	1000	389	65	No fault
487297	271385	179851	459845	333624	97074	No fault
388824	138072	483923	56377	336625	211156	No fault
308142	449556	190961	464427	256989	202072	No fault
441916	269710	7045	478904	116994	313584	No fault
362425	253480	104585	388782	404053	348857	No fault
138560	199740	245667	459335	189531	281963	No fault
362881	260366	218083	144747	239282	209	No fault
206	998	323	709	83	345	No fault
953	737	464	39	465	657	No fault
523	438	217	697	769	55	No fault
230	367	664	777	375	632	No fault
513	677	693	980	176	18	No fault
98	38	1	3	0	7	No fault
11	12	22	78	31	32	No fault
140	1	76	97	35	24	No fault
38	48	25	72	90	31	No fault
0	44	62	73	22	7	No fault
3	3	85	37	40	2481	No fault
7746	2016	6945	1443	7806	3307	No fault
1642	976	6804	6685	6790	1882	No fault
1585	4829	2572	1839	186	3231	No fault
7722	5145	1712	6242	1730	2973	No fault
7919	6490	1697	4115	3548	4910	No fault
7487	4463	2511	6973	2605	6531	No fault
2456	1381	7237	5040	4641	7365	No fault
5884	4880	2293	4776	2489	5147	No fault
2443	3422	6394	3000	7852	1797	No fault
4395	5201	2121	6788	6933	149	No fault
7613	1120	3393	4751	3363	2494	No fault
2366	1031	7025	108	5909	5272	No fault
5054	4144	6974	7020	4174	6354	No fault

### Training Stage

Firstly, the input data and target data are imported into the network. The gas composition was set as the input and the transformer incipient fault was set as the target. In this work, 100 input data consist of 6 types of gases were used while the fault, which was used as output can be classified into no fault, thermal fault, low intensity and high intensity. These data were categorised into training, validation and testing sets. The training set was 70% of total 100 data and 15% each for validation and test data.

In the training stage, a backpropagation algorithm is a generalised delta rule for feed-forward network with multiple of layers. This is due to its possibility to compute the gradient of each layer iteratively by using chain rule [27]. Generally, a sigmoid activation function is used because of its nonlinearity and compatibility with feed-forward backpropagation-learning algorithm to perform better. In this work, the Lavenberg-Marquart (LM) is used as the training function since it is fast, simple and robust algorithm. Thus, feed-forward backpropagation-learning algorithm was set as the network type for ANN architecture of this work.

By tuning the number of hidden layers, number of neurons and transfer function, the best parameters for ANN were selected with the highest accuracy, which equals to, *R*. A three-layer network which consists of two hidden layers and one output layer was used in this work. Although one hidden layer is enough for nonlinear mapping, a network with two hidden layer is the optimal in iteration number, accuracy and complexity compared to the network with one and three hidden layers. Moreover, three-layer network can overcome the problem of slow rate of training.

In developing the best ANN, the learning rate (LR) and momentum cost (MC) were varied from 0 to 0.9 to obtain the optimised value of LR and MC [28]. Since all parameter were varied heuristically, the problem of underfitting and overfitting network could occur. Overfitting occurs when the network is capable to memorise the network but cannot generalise the new data for network. To overcome the overfitting problem, early stopping technique was applied to develop better performance. The stop criterion is determined by comparing the mean square error of the training data while training with the data with a certain limit.

### Testing Stage

To test the trained network, a new set of data was simulated. The output of the new data set was simulated using the trained ANN. The best trained network shows that the simulated output agrees well with the target output. A regression coefficient, *R* is used to determine the performance of the trained network.

### Particle Swarm Optimisation (PSO)

PSO is a computational optimised technique which was invented by Kennedy and Eberthart in 1995 with the concept of bird flocking and fish schooling behaviour [29]. An optimisation problem can be formulated as a flock of birds fly across an area seeking for spot with abundant food. To find the optimised value, ANN is a time-consuming and difficult on computational process due to its heuristic characteristic. Hence, PSO is a better approach to find the optimised LR and MC values in ANN. In this work, MATLAB programming language was used to execute various PSO algorithms.

**Fig 2:**
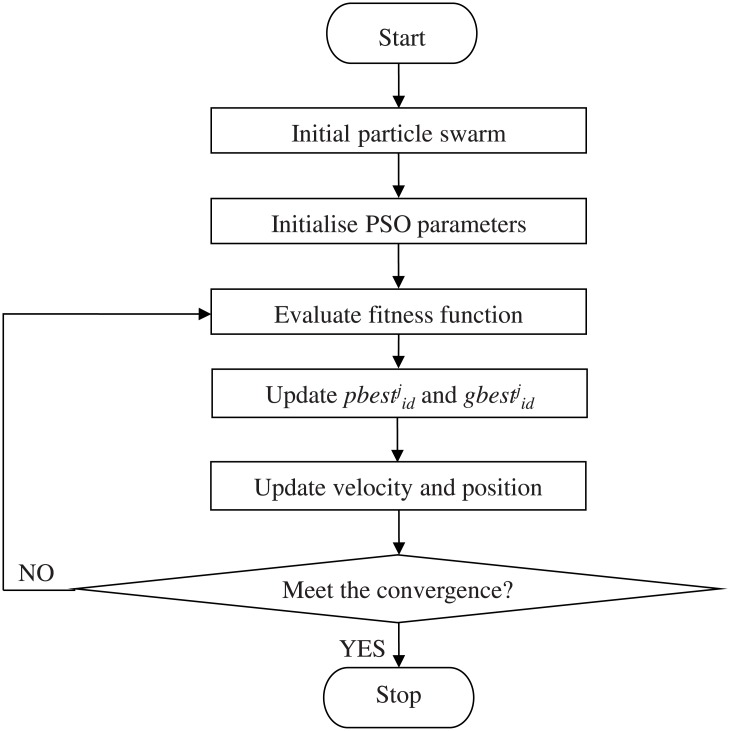
Flowchart of PSO technique.

In PSO, the population-based search is used to achieve the optimised objective function. Firstly, the potential solution known as particles is initialised randomly and explores in a dimension, *d* search region. With the strategy of each particle updates its velocity and position, the particle swarm will move nearer to the region with higher object value. The flowchart of PSO technique is shown in Fig 2. The steps in PSO are explained as follows [30]:

### Step 1: Initialisation

The swarm is initialised by setting the position and the velocity of particles randomly.

### Step 2: Evaluate fitness function

The fitness value for each particle with updated position and velocity is calculated.

### Step 3: Update *pbest* ^*j*^_*id*_ and *gbest* ^*j*^_*id*_

The fitness value of each particle is compared with personal best, *pbest* ^*j*^_*id*_. If the new fitness value is better than the *pbest* ^*j*^_*id*_, this value will be set as *pbest* ^*j*^_*id*_ and the current position of particle, *X* ^*j*^_*id*_. Among the entire particle, the best fitness value will be set as *gbest* ^*j*^_*id*_ or global best value.

### Step 4: Update velocity and position

The velocity and position of all particles are updated using

$$V_{id}{}^{j+1}=wV_{id}{}^{j}+c_{1}r_{1}(pbest_{id}{}^{j}-X_{id}{}^{j})+c_{2}r_{2}(gbest_{id}{}^{j}-X_{id}{}^{j})$$ (1)

$$X_{id}{}^{j+1}=X_{id}{}^{j}+V_{id}{}^{j+1}$$ (2)

where

$V_{id}{}^{j+1}$ = updated velocity of particle i in dimension *d* search region

$V_{id}{}^{j}$ = velocity of particle *i* at iteration *j*

$X_{id}{}^{j+1}$ = updated position of particle *i* in dimension *d* search region

$X_{id}{}^{j}$ = position of particle *i* at iteration *j*

*c*_1_, *c*_2_ = acceleration factors

*r*_1_, *r*_2_ = random constant between 0 and 1

*w* = inertia weight

$$=w_{\text{max}}-\left(\frac{w_{\text{max}}-w_{\text{min}}}{iteration_{\text{max}}}\right)\left(iteration\right)$$

*w*_*max*_ = maximum weight

*w*_*min*_ = minimum weight

### Step 5: Meet the end condition

Steps 2–4 are repeated until the stopping criterion is fulfilled such that the best fitness value is achieved or the number of iteration has been reached to its maximum. In this work, *c*_1_ and *c*_2_ were both set as 0.7, *w*_*max*_ and *w*_*min*_ were set as 0.9 and 0.4 the iteration was set as 100. These are the common values used in most of the PSO algorithms.

### Iteration particle swarm optimisation (IPSO)

PSO is easy to trap in the local minimum. Thus, improved PSO algorithm has been suggested for better performance, called as iteration PSO (IPSO) [31]. In IPSO, the best iteration, *I* ^*j*^_*best*,*d*_ is employed to improve the basic PSO performance in term of accuracy. The equation for the velocity in Eq (1) is modified as follows [32]:

$$V_{id}{}^{j+1}=wV_{id}{}^{j}+c_{1}r_{1}(pbest_{id}{}^{j}-X_{id}{}^{j})+c_{2}r_{2}(gbest_{id}{}^{j}-X_{id}{}^{j})+c_{3}r_{3}(I_{best,d}{}^{j}-X_{id}{}^{j})$$ (3)

where

$I_{best,d}{}^{j}$ = best fitness value which obtained by any particle in iteration *j*

*c*_3_ = weight of stochastic acceleration term to attract each particle toward $I_{best,d}{}^{j}$

$$c_{3}=c_{1}[1-\text{exp}(-c_{1}\times (iteration))]$$ (4)

### Evolutionary Particle Swarm Optimisation (EPSO)

EPSO is an optimisation technique which combines the concept of evolutionary strategies and PSO. The main advantage of EPSO is that the search of particle would not be focused on the region of global best fitness value, but the optimum may be in the neighbourhood if the optimal value has not been found. The concept of duplication, mutation and reproduction is employed in EPSO. For duplication, each particle is duplicated. Next, each particle with mutated weight w* will reproduce an offspring by abiding the particle movement rule. The equation for the velocity in Eq (1) is modified as follows [33]:

$$V_{id}{}^{j+1}=w_{i0}{}^{*}V_{id}{}^{j}+w_{i1}{}^{*}(pbest_{id}{}^{j}-X_{id}{}^{j})+w_{i2}{}^{*}(gbest_{id}{}^{*}-X_{id}{}^{j})$$ (5)

where

$gbest_{id}{}^{*}=gbest_{id}{}^{j}+\tau \prime N(0,1)$ is the mutated global best position

$w_{ik}{}^{*}=w_{ik}+\tau N(0,1)$ is the mutated weight

*τ* and *τ*’ = learning parameter

*N*(0,1) = a random variable with Gaussian distribution with 0 mean and variance of 1

In this work, *τ* and *τ*’ were varied from 0.1 to 0.9 until the best output from the ANN was achieved. It was found that the most suitable value for both *τ* and *τ*’ is 0.3.

## Diagnosis Results

### Fault prediction using IEC 60599 method

100 data were used in this work and classified into electrical fault, thermal fault and no fault with 32 cases, 16 cases and 50 cases respectively. IEC 60599 method was used to predict the fault by using the gas ratio. The results are tabulated in Table 2. Comparing the indicated fault with the actual fault occurred, IEC 60599 achieve 70% correct prediction of the transformer fault.

**Table 2:** Comparison of the indicated result by IEC 60599 with the actual result.

Fault type	Actual fault	IEC 60599 method
**Electrical Fault**	32	31
**Thermal Fault**	16	15
**No fault**	50	24
**Correct prediction (%)**	100%	70%

### Fault prediction using ANN alone

The simulations were performed by using different number of hidden layer, number of neurons, learning rate (LR) and momentum constant (MC) in the ANN. The process of finding the best ANN model is as follows:

#### 1. Variation of number of neurons in hidden layer (HL)

Before determining the parameters of LR and MC, typical values of LR and MC were used as 0.05 and 0.95 respectively. The training function and learning function used were Lavenberg-Marquart (TRAINLM) and Gradient Descent with momentum weight and bias learning function (LEARNGDM). The number of neurons in HL1 was increased from 2 to 20 with a step of 2 by keeping other parameters constant. The transfer functions of HL1 and HL 2 were logsig-logsig while the transfer function for output layer was pure-linear (PURELIN).

The next stage was increasing the number of neurons in HL2 from 2 to 20 with a step of 2 by keeping other parameters constant. 100 set of data with different number of neurons in HL1 and HL2 were tested. The ANN model with the highest *R* value was selected as shown in Table 3. The number of neurons for HL1 and HL2 was selected as 4 and 10 respectively since higher number of neurons than these values do not yield any further improvement in the network performance.

**Table 3:** Properties of the selected ANN using default LR and MC.

ANN parameters	Type/Value
**Training Function**	TRAINLM
**Learning Function**	LEARNGDM
**Number of Neurons in HL1**	4
**Number of Neurons in HL2**	10
**Transfer function**	Logsig-logsig
**LR**	0.05
**MC**	0.95
***R***	0.9451
**% of correct fault prediction**	94%

#### 2. Variation of LR and MC values

By keeping MC constant, the value of LR was increased from 0 to 0.9 with a step of 0.01. For each LR, the value of MC was increased from 0 to 0.9 with a step of 0.1. The outcome from this neural network is shown in Table 4.

**Table 4:** The properties of selected ANN after tuning LR and MC.

ANN parameters	Type/Value
**Training Function**	TRAINLM
**Learning Function**	LEARNGDM
**Number of Neurons in HL1**	4
**Number of Neurons in HL2**	10
**Transfer function**	Logsig-logsig
**LR**	0.01
**MC**	0.9
***R***	0.9505
**% of correct fault prediction**	95%

### Fault prediction using ANN with Particle Swarm Optimisation (PSO)

In order to find the optimised value of LR and MC in ANN, PSO was employed. Since there are two parameters need to be optimised, LR and MC were defined as *particle1* and *particle2* respectively in the PSO. Using PSO, the optimised result for LR, MC and the best position were obtained. The simulation process of PSO was run for 20 times to test the robustness of the method. Next, the ANN was trained with the data obtained from the PSO. The results from the ANN-PSO method are shown in Table 5.

**Table 5:** Properties of the selected ANN combined with PSO.

ANN parameters	Type/Value
**Training Function**	TRAINLM
**Learning Function**	LEARNGDM
**Number of Neurons in HL1**	4
**Number of Neurons in HL2**	10
**Transfer function**	Logsig-logsig
**LR**	0.09
**MC**	0.5579
***R***	0.9578
**% of correct fault prediction**	96%

### Fault prediction using ANN combined with iteration PSO (IPSO)

The ANN model was also combined with IPSO. The IPSO technique was used to obtain the optimised values of LR and MC. Similar process as ANN-PSO method was repeated for ANN-IPSO method. The optimised value of LR and MC, *R* value and the percentage of correct fault prediction obtained from this method are shown in Table 6.

**Table 6:** Properties of the selected ANN combined with IPSO.

ANN parameters	Type/Value
**Training Function**	TRAINLM
**Learning Function**	LEARNGDM
**Number of Neurons in HL1**	4
**Number of Neurons in HL2**	10
**Transfer function**	Logsig-logsig
**LR**	0.09
**MC**	0.6806
***R***	0.9644
**% of correct fault prediction**	97%

### Fault prediction using ANN combined with evolutionary PSO (EPSO)

The ANN model was also combined with EPSO. The EPSO technique was used to obtain the optimised values of LR and MC. Similar process as ANN-PSO method was repeated for ANN-EPSO. The results obtained from the ANN-EPSO method are shown in Table 7.

**Table 7:** The properties of selected ANN combined with EPSO.

ANN parameters	Type/Value
**Training Function**	TRAINLM
**Learning Function**	LEARNGDM
**Number of Neurons in HL1**	4
**Number of Neurons in HL2**	10
**Transfer function**	Logsig-logsig
**LR**	0.09
**MC**	0.2429
***R***	0.9769
**% of correct fault prediction**	98%

### Comparison between ANN, ANN-PSO, ANN-IPSO and ANN-EPSO methods

In order to identify the best technique to predict the transformer incipient fault, the ANN alone, ANN combined with PSO, IPSO and EPSO techniques were compared in terms of *R* value, percentage of correct fault prediction and convergence rate. From Table 8, it can be seen clearly that the ANN-EPSO technique yields the highest *R* value and percentage of correct fault prediction, followed by the ANN-IPSO technique, ANN-PSO technique and finally the ANN only. All of these methods yield higher correct prediction of transformer incipient fault than the existing DGA method, which is IEC method. *R* value for the ANN alone is the least because ANN is heuristic in nature. In EPSO technique, the value of LR and MC obtained underwent duplication, mutation and reproduction. Thus, ANN with EPSO yields the best results in identifying the transformer incipient fault compared to ANN, ANN-PSO and ANN-IPSO techniques.

**Table 8:** Comparison of the results between different techniques.

Parameters	ANN	ANN-PSO	ANN-IPSO	ANN-EPSO
**LR**	0.01	0.09	0.09	0.09
**MC**	0.9	0.5579	0.6806	0.2429
***R***	0.9505	0.9578	0.9644	0.9769
**% of correct prediction**	95%	96%	97%	98%
**Convergence (iteration)**	-	38	20	11

The proposed methods were also compared with the existing methods that have been reported in literature. Table 9 summarises the comparison results. From this table, it can be seen that the proposed ANN-EPSO yields the highest percentage of correct transformer identification. Although EPSO is simple and easy to be implemented, it yields the best result when combined with ANN. In EPSO, the search of particle would not be focused on the region of global best fitness value, but the optimum value may be in the neighbourhood if the optimal value is not found.

**Table 9:** Comparison of the proposed methods with previous methods.

Method	Accuracy (%)
**ANN**	95
**ANN-PSO**	96
**ANN-IPSO**	97
**ANN-EPSO**	98
**ANNEPS [4]**	90.95
**EPANN [28]**	95
**Fuzzy Logic [15]**	89
**Immune Neural Network [7]**	86.3
**Rough Set Theory [6]**	81.25
**GP-KNN [5]**	92.11
**Support Vector Machine (SVM) [10]**	92
**Genetic wavelets network (GWN) [8]**	96.19
**Self-organizing polynomial network (SOPN) [9]**	97.68

**Fig 3:**
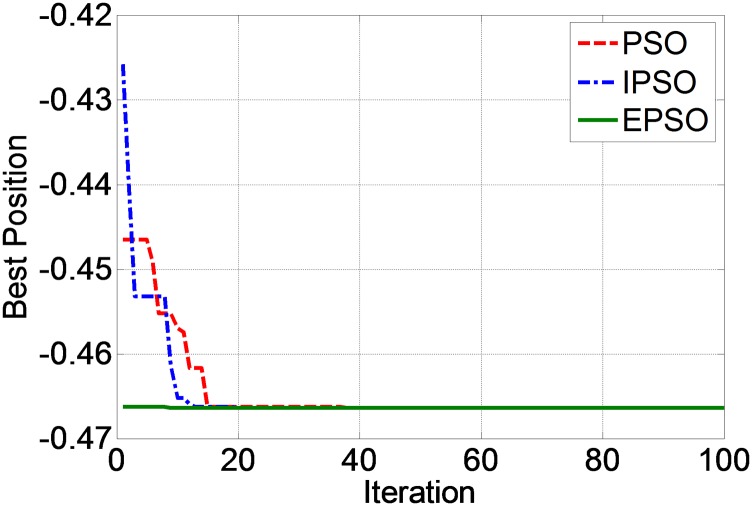
Best position vs. iteration for PSO, IPSO and EPSO techniques.

**Fig 4:**
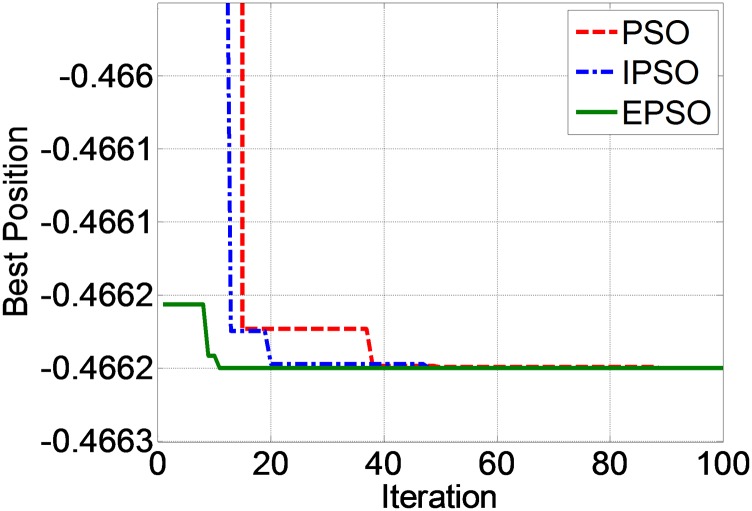
Best position vs. iteration for PSO, IPSO and EPSO techniques (closer view).

A graph of the best position against iteration for ANN with each PSO technique is shown in Fig 3. From this figure, it can be seen that the convergence rate of EPSO is the fastest, followed by the IPSO and PSO. Fig 4 shows a closer view of Fig 3 at the convergence zone. From this figure, PSO, IPSO and EPSO converged to the best position at -0.46625. The iteration where each method converges to the best position is shown in Table 8.

## Conclusions

In transformer incipient fault recognition, the relationship between gas type and fault is nonlinear. This causes problem in the convergence rate and oscillation in artificial neural network (ANN). The parameters of the ANN must also be properly tuned in order to obtain the best performance of the network. To overcome these problems, in this work, a method of combination of artificial neural network (ANN) and various particle swarm optimisation (PSO) techniques to predict transformer incipient fault has been successfully proposed. In this method, the ANN was used to identify the transformer incipient fault and various techniques of PSO were applied to optimise the performance of the ANN. The performance of various PSO techniques in combination with ANN was compared with the existing DGA method, ANN alone and previously reported work to identify the best method for transformer incipient fault prediction. It was found that the method of combination of ANN with evolutionary PSO (EPSO) yields the best performance in the transformer fault prediction compared to the existing DGA method and previously reported works. Hence, this method can be proposed as one of the solutions in the field diagnosis of transformer incipient fault.
